# Predictors of Virological Response to a Combination Therapy with Pegylated Interferon Plus Ribavirin Including Virus and Host Factors

**DOI:** 10.1155/2010/703602

**Published:** 2010-09-05

**Authors:** Namiki Izumi, Yasuhiro Asahina, Masayuki Kurosaki

**Affiliations:** Department of Gastroenterology and Hepatology, Musashino Red-Cross Hospital, Kyonancho 1-26-1, Musashinoshi, 180-8610 Tokyo, Japan

## Abstract

A combination therapy with pegylated interferon (PEG-IFN) plus ribavirin (RBV) has made it possible to achieve a sustained virological response (SVR) of 50% in refractory cases with genotype 1b and high levels of plasma HCVRNA. Several factors including virus mutation and host factors such as age, gender, fibrosis of the liver, lipid metabolism, innate immunity, and single nucleotide polymorphism (SNPs) are reported to be correlated to therapeutic effects. However, it is difficult to determine which factor is the most important predictor for an individual patient. Data mining analysis is useful for combining all these together to predict the therapeutic effects. It is important to analyze blood tests and to predict therapeutic effects prior to initiating treatment. Since new anti-HCV agents are under development, it will be necessary in the future to select the patients who have a high possibility of achieving SVR if treatment is performed with standard regimen.

## 1. Progress in Virological Response in the Difficult-to-Treat Patients with Genotype 1 Hepatitis C Virus (HCV) Infection and Factors Correlated to the Efficacy

Recently, the average age of the patients with chronic hepatitis C has been increasing in Japan. Incidence of hepatocellular carcinoma (HCC) in the elderly patients with chronic hepatitis C (65 years or older) has demonstrated to be higher than younger ones when adjusted by the stage of hepatic fibrosis [1]. In Japan, refractory cases with genotype 1b and high HCVRNA levels are seen in as high as 70 percent of chronic hepatitis C patients. The outcome of conventional IFN monotherapy has been an SVR response of 3%–5% after 6 months of treatment in genotype 1b and high HCVRNA patients [2, 3], and virus mutation such as interferon sensitivity-determining region (ISDR) is shown to be correlated with the virological response [2]. The association of ISDR mutations and virological response to IFN monotherapy was denied in an Italian study [4]; however, it was confirmed by a Chinese study [5] and an international meta-analysis [6].

However, pegylated IFN (PEG-IFN) extends the duration of therapy and reduces adverse effects, and for this reason, PEG-IFN has become the cornerstone of therapy. Furthermore, by the combination therapy with PEG-IFN and ribavirin (RBV), the rate of SVR has dramatically improved. Even in the patients with genotype 1b and high HCVRNA level, SVR rate reaches as high as 40%–50%, thereby improving the therapeutic effects both in Western countries [7, 8] and in Japan [9, 10].

 It is important to predict the rate of achieving SVR in the individual patient, before initiating treatment. Both virus- and host-related elements have been reported as factors correlated to therapeutic effects of combination therapy [11–13]. A particular focus has been placed on virus mutations, age, gender, fibrosis of the liver, lipid metabolism, and degree of fatty metamorphosis of the liver.

**Figure 1:**
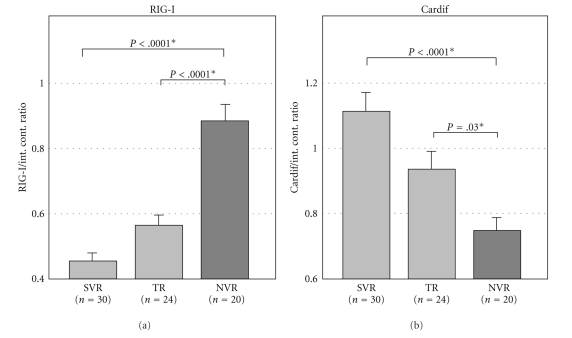
Expression of genes correlated to the intrahepatic innate immunity and virological response to PEG-IFN alpha-2b and RBV combination therapy. Open column indicates SVR (*n* = 30), gray column indicates TR (*n* = 24), and closed column indicates NVR (*n* = 20). Error bars indicate the standard error. The *P* values were analyzed by the Kruskal-Wallis test. Expression of Rig-I was significantly higher in NVR than in SVR patients, and Cardif expression was higher in SVR than in NVR. The figure was cited from [8].

 Among these factors related to PEG-IFN and RBV, innate immunity has been shown to be correlated in virological response. Asahina et al. reported that liver biopsies were performed before the PEG-IFN and RBV combination therapy to examine the correlation between the gene expression involved in innate immunity and the therapeutic effects, and in the patients in whom RIG-I expression is high and the expression of Cardif, an adaptor gene, is low, it was found that there are many nonresponders (NVRs) in which HCVRNA does not become negative during the course of treatment [13]. It was therefore revealed that there are many NVRs among the patients in whom the ratio of RIG-I to Cardif in liver tissue is high and that this ratio is low in the SVR patients. Based on these findings, it has become clear that innate immunity is correlated to therapeutic effects (Figure 1).

 Furthermore, it was recently discovered that a single nucleotide polymorphism (SNP) of the host gene IL28B is significantly involved in the therapeutic response to the PEG-IFN and RBV combination therapy [14, 15]. The possibility of becoming an NVR is high in cases of the minor allele carriers of IL28B. However, it is not possible to routinely measure an SNP of IL28B in the clinical setting. In this paper, factors which can actually be used in real clinical practice are discussed for the prediction of the efficacy of PEG-IFN and RBV combination therapy.

## 2. Amount of HCVRNA

 In the patients with chronic hepatitis C, it is not possible to directly measure the amount of virus, and the amount of HCVRNA is measured instead. Currently, a real-time PCR method which has an advantage of wide range and high sensitivity is utilized, and measurements can be taken from a single blood sample of a very small amount, that is, 1.2 log copies/ml, to a very large amount, that is, 8 log copies/ml. This method has a higher level of sensitivity than the conventional Amplicor monitor test and can therefore detect HCVRNA even if only a very small amount exists in the plasma. If the amount of HCVRNA in plasma is less than the range of sensitivity of the real-time PCR method, it is recorded as undetectable level. If the indication is “less than 1.2 log copies/ml of HCVRNA”, it means that a very small amount of HCVRNA exists in the plasma. Since the indication of the real-time PCR method is based on log counts, a decrease of 1.0 in the numerical value means that the amount of HCVRNA has decreased to 1/10. With the application of this real-time PCR method, it has become possible to measure amounts of HCVRNA up to 8 log copies/ml, and it has also become possible to predict the efficacy before treatment and to monitor appropriately the reactivity during the course of treatment. However, in the patients in whom a PEG-IFN and RBV combination therapy is performed, SVR can be acquired even when the amount of virus prior to the treatment is quite large. It is therefore difficult to predict the virological response solely from the amount of HCVRNA before starting the treatment. Once treatment has commenced, at what week HCVRNA becomes negative is important for the prediction of therapeutic effects, and this serves as a parameter for deciding the duration of treatment [16].

Measuring the rate of viral clearance from serum is helpful for predicting the likelihood of a response to PGEIFN and RBV and useful for determining the optimal duration of therapy if the patients start to receive the treatment [17]. In the AASLD practice guideline, response-guided therapy is highly recommended [18]. In two nationwide registration trials conducted in Japan [9, 10], the SVR rate was high, from 76% to 100%, in patients whose HCVRNA was cleared rapidly from serum by week 4 (rapid virological response; RVR), and 71% to 73% in patients who achieved undetectable HCVRNA from week 5 to week 12 (early virological response; EVR). In contrast, the SVR rate in patients with late clearance of HCVRNA from week 13 to week 24 was low at 29% to 36%. No patients without clearance of HCVRNA by week 24 achieved SVR. 

 The strategy of extending therapy in patients with delayed virological responses, defined as clearance of HCV-RNA between weeks 12 and 24, was evaluated in five studies [19–23]. These results cannot be compared directly with each other because of the heterogeneous study populations, differences in the baseline characteristics, and the different regimens utilized amongst them. Nevertheless, the results showed a trend toward a higher SVR rate by extending therapy from 48 to 72 weeks in patients with delayed virological response.

## 3. Viral Mutations in Core and NS5A Region

**Figure 2:**
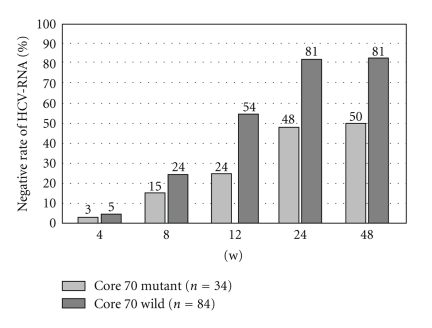
Comparison of aa70 mutations in the HCV core region and the rate of HCVRNA becoming negative during the course of treatment. Compared with the wild type, among the patients of aa70 mutations, there were fewer patients in whom HCVRNA had become negative during the course of treatment.

 In the patients with genotype 1b HCV infection, the mutations in aa70 and aa91 in the core region have been shown to correlate with early virological response (EVR) during PEG-IFN and RBV combination treatment [11]. If aa70 in the core region is mutated to anything other than arginine and aa91 to anything other than leucine, it is difficult to achieve EVR, and it is thus highly possible that such cases will become nonresponders. The examination results at our institution including 292 patients with genotype 1b infection demonstrated that, in the cases with mutations in aa70 in the core region, the rate of HCVRNA becoming negative during the course of combination treatment was low compared to the wild type of aa70 (Figure 2). However, core aa70 mutation is shown to have quasispecies detectable by cloning, and 70Q clone was positively selected during combination treatment with PEGIFN and RBV [24].

 Furthermore, Enomoto et al. reported that the patients with 4 or more amino acid mutations were observed in interferon sensitivity-determining region (ISDR) within NS5A region [2]; SVR rate is higher than 90% by IFN monotherapy, and SVR is less than 10% in the patients with no mutation in ISDR. It has also been reported that, in PEG-IFN and RBV combination therapy, the number of ISDR mutations is involved in the SVR [12].

**Figure 3:**
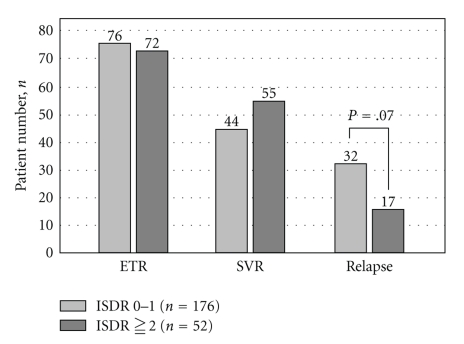
Number of ISDR substitutions and the comparison of virological response, SVR, and relapse at the end of the treatment. Compared with the patients with 2 or more sites of substitutions, the rate of SVR was lower and the rate of relapse was higher in the patients in whom there were fewer substitutions in ISDR, that is, 0 or 1 sites.

We analyzed the relationship between virological response and ISDR mutations in the patients with genotype 1b infection treated by PEG-IFN alpha-2b and BRV combination therapy. In the patients with 0 or 1 ISDR mutation, even if the rate of HCVRNA becoming negative at the end of treatment was the same, the rate of SVR would be lower compared with the patients having 2 or more mutations (Figure 3). This demonstrates that there is a higher incidence of relapse after the end of treatment in the patients with 0 or 1 ISDR mutation.

Enomoto and Maekawa reported that mutations both in NS5A-ISDR (interferon sensitivity-determining region) and core 70Q substitution are associated with no early viral response during PEGIFN and RBV combination therapy [25]. Association of core aa70 substitution and mutations in NS5A region is confirmed to be associated with viral response by PEGIFN and RBV combination therapy in a Japanese multicenter cooperative study [26]. The number of mutations in the interferon sensitivity-determining region was shown to be associated with the viral response to PEGIFN and RBV combination treatment not only in Japan [27], but also in Tunisia [28].

Recently, a consensus has been established that mutations in aa70 in the core region are important for the prediction of HCVRNA becoming negative during the early course of treatment, and the number of ISDR mutations is important for the prediction of relapse after the end of treatment.

## 4. Adherence

 It has been confirmed that it is important to ensure 80% or more of the scheduled dose of both PEG-IFN and BBV in order to improve the rate of SVR, and together with the duration of treatment, the 80 · 80 · 80 rule has been established. However, Schiffman et al. recently reported that the dose of PEG-IFN in the initial stage of administration is important and that, if a sufficient dose of PEG-IFN is administered, then 60% or more of the RVB dose would be enough [29]. It is therefore of primary importance to ensure the dose of PEG-IFN. 

 In Japan, the average age of patients with chronic hepatitis C is increasing, and achieving good adherence is difficult in many patients. Consequently, the rate of SVR is low in elderly patients. How to improve the rate of SVR in elderly patients is an important issue. With regard to the dose of RBV, reducing the RBV dose based on the calculation of the total body clearance (CL/F) has been proposed to be useful for decreasing the discontinuation and improving the rate of SVR. Although there is no consensus on an appropriate dose of PEG-IFN in elderly patients, if the initial dose is set lower than the usual dose, discontinuation would be reduced. Thus, it is necessary to investigate whether such an initial dose would improve the rate of SVR.

 Recently, the risk of hemolytic anemia was clearly demonstrated to correlate with ITAP gene SNP [30]. The predictive implication should be analyzed prospectively in clinical practice.

## 5. Host Factors

Zeuzem et al. described the factors related to the less response to interferon-based therapy, and he showed that several host factors such as older age, race, and obesity are responsible factors for the poor response to IFN [31]. Recently, insulin resistance which was examined by homeostasis model assessment index (HOMA-IR) was shown to be associated with a lower rate of SVR, and body mass index (BMI) was not identified as a significant factor for the poor response to PEGIFN and ribavirin combination therapy [32]. Insulin resistance was confirmed as a related factor to the nonresponse to interferon-based treatment [33]. However, Charlton et al. reported that obesity itself is an associated factor for decreased efficacy of interferon-based therapies, and they discussed the possible mechanism [34], and obesity was shown to be associated with the increased enhancement of suppressor of cytokine signaling (SOCS) family in the hepatocytes [35].

## 6. Data Mining Analysis

 Both virus- and host-related factors are correlated to therapeutic effects of PEG-IFN and RBV. One important question is which of these factors should be focused on in order to predict the therapeutic effects in an individual patient. In addition, in each individual patient, the host and virological factors are different. It is therefore difficult to predict the virological response in each case before treatment. Furthermore, although it is important to predict the relapse rate when HCVRNA becomes within an undetectable level in an individual patient, prediction of the rate of SVR including virological and host factors and adherence to the treatment has never been carried out in an individual patient.

 A data mining analysis is the process of analyzing a large amount of data by a computer in order to develop an algorithm. Conventional statics have been used to examine certain hypothesis. Data mining is superior in that it can set an algorithm, using a computer, based on a large amount of data without a hypothesis. 

**Figure 4:**
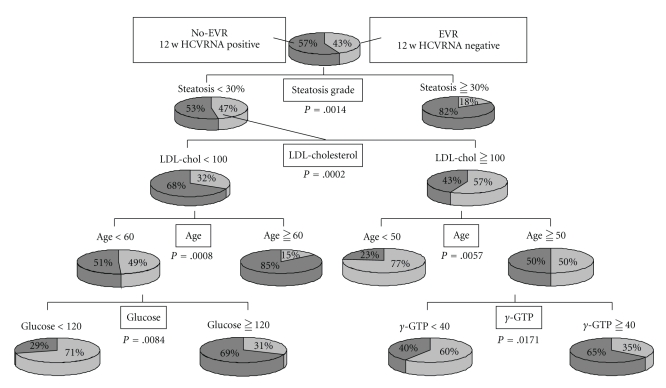
HCVRNA negative (EVR) algorithm at 12th week from data mining analysis of PEG-IFN alpha-2b plus RBV combination in the genotype 1b and high levels of HCVRNA. Both virological and host factors were evaluated by data mining analysis software from SPSS. This figure was cited from [36].

 We therefore conducted at our institute a data mining analysis of the patients with genotype 1b infection having high levels of HCVRNA to whom a PEG-IFN alpha-2b and RBV combination therapy was administered to investigate whether by the 12th week after the commencement of treatment HCVRNA became negative (EVR) (Figure 4) [36]. The most important factor for the prediction of EVR was the steatosis of the liver: when steatosis was observed in 30% or more of hepatocytes, EVR was found to be difficult to achieve. In the patients in whom steatosis was not severe, the second most important factor was the serum LDL cholesterol value. While the rate of EVR was 57% in the patients in whom this value was 100 mg/ml or above, the rate of EVR was 32% in the patients in whom the LDL cholesterol was less than 100 mg/dl. 

 The higher the LDL cholesterol value, the earlier the HCVRNA became negative. Among the patients with low LDL cholesterol values, while the rate of EVR was 15% in patients 60 years of age or older, the rate was high in the patients under the age of 60 years old, that is, 49%. Among patients under the age of 60, the rate of EVR was low, that is, 31%, in patients with a blood glucose level of 120 mg/dl or above whereas EVR was achieved in 71% of the patients with a blood glucose level of less than 120 mg/dl (Figure 4).

 On the other hand, in the patients with high LDL cholesterol values, the next most important factor was age. While the rate of EVR was 50% in patients 50 years of age or older, EVR was achieved in 77% of the patients under the age of 50. Among patients of 50 years of age or older, the next most important factor was the gamma GTP value. While the rate of EVR was 35% in the patients in whom gamma GTP was 40 IU/L or above, EVR was achieved in 60% of the patients where the value was less than 40 IU/L.

**Figure 5:**
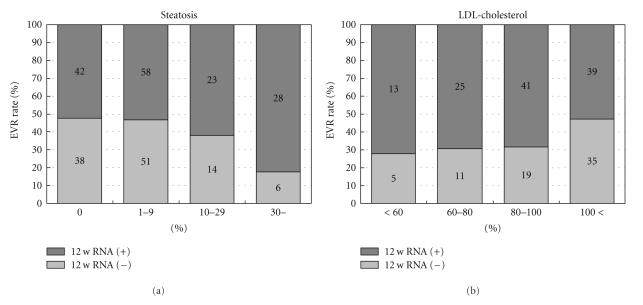
The rate of EVR in the patients with genotype 1b and high levels of HCVRNA, based on fatty deposition in the liver (a), and the LDL cholesterol value (b), respectively. EVR was highly achieved in the patients with less steatosis in the liver, and in those with high serum LDL-cholesterol levels. This is univariate analysis, and cited from [36].

 We therefore compared these factors based on the original data. A univariate comparison of the fatty infiltration of the liver and the rate of EVR demonstrated that the rate of EVR was higher when the steatosis of the liver was less severe (Figure 5(a)). In addition, a comparison of the LDL cholesterol value and the rate of EVR demonstrated a significant correlation, confirming that the higher the LDL cholesterol value, the higher the rate of EVR (Figure 5(b)). Therefore, it was also proposed by the results of univariate analysis of each factor extracted from the data mining analysis that these factors were correlated to the rate of EVR.

 From these observations, it is likely to improve the viral response to PEGIFN and ribavirin by reducing steatosis of the liver through daily walking or abstinating alcohol intake or by refraining from high-fat diet. 

 By utilizing data mining, it is therefore possible to assess virus- and host-related factors together and to predict the virological response in each patient, and thereby clinically useful information can be obtained. The algorithm should be validated including a large number of the patients.
